# Heme biomolecule as redox mediator and oxygen shuttle for efficient charging of lithium-oxygen batteries

**DOI:** 10.1038/ncomms12925

**Published:** 2016-10-19

**Authors:** Won-Hee Ryu, Forrest S. Gittleson, Julianne M. Thomsen, Jinyang Li, Mark J. Schwab, Gary W. Brudvig, André D. Taylor

**Affiliations:** 1 Department of Chemical and Environmental Engineering, Yale University, 9 Hillhouse Avenue, New Haven, Connecticut, USA; 2 Department of Chemical and Biological Engineering, Sookmyung Women's University, 100 Cheongpa-ro 47-gil, Yongsan-gu, Seoul, Republic of Korea; 3 The Nature Conservancy, Arlington, Virginia, USA; 4 Materials Chemistry Department, Sandia National Laboratories, 7011 East Avenue, Livermore, California 94550, USA; 5 Department of Chemistry, Yale University, 225 Prospect Street, New Haven, Connecticut, USA

## Abstract

One of the greatest challenges with lithium-oxygen batteries involves identifying catalysts that facilitate the growth and evolution of cathode species on an oxygen electrode. Heterogeneous solid catalysts cannot adequately address the problematic overpotentials when the surfaces become passivated. However, there exists a class of biomolecules which have been designed by nature to guide complex solution-based oxygen chemistries. Here, we show that the heme molecule, a common porphyrin cofactor in blood, can function as a soluble redox catalyst and oxygen shuttle for efficient oxygen evolution in non-aqueous Li-O_2_ batteries. The heme's oxygen binding capability facilitates battery recharge by accepting and releasing dissociated oxygen species while benefiting charge transfer with the cathode. We reveal the chemical change of heme redox molecules where synergy exists with the electrolyte species. This study brings focus to the rational design of solution-based catalysts and suggests a sustainable cross-link between biomolecules and advanced energy storage.

## Abstract (web summary)

Natural biomolecules including heme-like porphyrins can be applied as sustainable chemical catalysts in lithium-oxygen batteries. Here, the authors show that the heme molecule can function as a soluble redox catalyst and oxygen shuttle for efficient oxygen evolution in non-aqueous lithium-oxygen cells.

## Graphical abstract

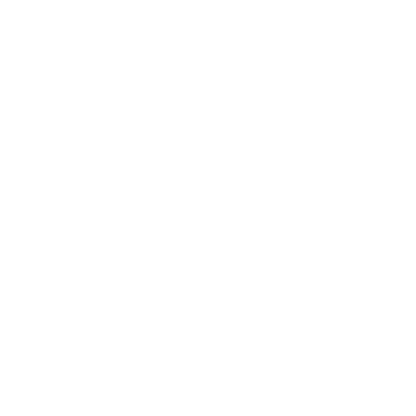

The goal of developing next-generation energy storage systems ‘beyond lithium-ion' will be achieved by employing novel, efficient and sustainable battery chemistries^1^,^2^. Lithium-oxygen (Li-O_2_) batteries utilizing lightweight and abundant reactants have drawn significant interest due to their high-energy density, two to three times greater than lithium-ion cells^3^,^4^,^5^,^6^. The most common, non-aqueous variety of Li-O_2_ battery operates via surface reactions, which involve the formation and evolution of solid lithium oxides (2Li^+^+O_2_+2e^−^↔Li_2_O_2_, *E*°=2.96 V versus Li/Li^+^)^7^. However, the evolution reaction, which dictates cell reversibility, suffers from large (∼1 V) overpotentials and impeded kinetics due to poor electron conduction in the solid products^8^. Efforts have been made to develop various solid-state catalysts (that is, noble metals, metal oxides) decorated on oxygen electrodes, yet catalytic sites are easily deactivated by the precipitation of solid products and consequently electron transfer at the solid/solid interface is too slow^9^,^10^,^11^,^12^,^13^. In this regard, new catalyst chemistries should be considered to alleviate electrode deactivation and offer continuous catalytic function^14^,^15^. Incorporating catalytic molecules directly into electrolytes can improve the accessibility of these species to interfacial products and the electrode surface^16^,^17^. Moreover, redox molecules can mediate the efficient transport of electrons between the insulating products and the oxygen electrode during oxygen evolution, reducing ohmic losses^18^,^19^,^20^. Potential redox molecules should offer: (i) good solubility and facile diffusion in Li^+^ containing electrolytes, (ii) fast electron transfer with the oxygen electrode, (iii) oxygen-complexing ability to increase electrolyte oxygen solubility, and (iv) natural abundance with a low environmental impact. A few redox molecules (for example, Li iodide (LiI), tetrathiafulvalene (TTF), iron phthalocyanine (FePc), (2,2,6,6-tetramethylpiperdin-1-yl)oxyl (TEMPO), and tris[4-(diethylamino)phenyl]amine (TDPA)) have been investigated for Li-O_2_ systems and have shown promise for this approach^16^,^18^,^21^,^22^,^23^. However, most of these redox molecules only operate via simple electron transfer reactions and do not have an established oxygen-complexing ability.

Intermittent attempts to introduce biomolecules into energy storage and conversion devices have demonstrated some success with flow batteries, fuel cells and solar cells^24^,^25^,^26^. Using biomolecules extracted from natural supplies can theoretically address the global demand for sustainable and ubiquitous chemical catalysts. Recyclable bio-wastes (for example, blood waste, dietary trash, natural enzymes), may be employed for the environmentally friendly and cost-effective production of catalysts^27^. The heme molecule, a porphyrin with an Fe ion centre and a cofactor in the blood protein hemoglobin, has been highlighted in diverse applications involving oxygen transport^28^,^29^,^30^. The heme metalloenzyme, acting as an oxygen shuttle and reservoir, exhibits auto-oxy/deoxygenation properties, responding to external environments where its Fe ion centre promotes chemical and electrochemical redox^31^,^32^,^33^,^34^. The Fe ion centre of the heme is bound to four pyrrole nitrogens, leaving an open axial coordination site that can bind dioxygen (Fig. 1a)^35^. The reversible redox characteristics (Fe^3+^/Fe^2+^ couple) facilitate catalysis by rapid electron transfer to/from Fe ions in heme^36^,^37^,^38^. While the redox activity of heme has been widely established in aqueous environments (∼1 V)^34^,^37^, the behaviour of heme molecules in non-aqueous lithium electrolytes remains poorly understood. Unlike the conventional oxygen-evolving reaction in water, which is a liquid to gas phase process, oxygen evolution in Li-O_2_ cells occurs via a solid (that is, LiO_2_, Li_2_O_2_) to gas phase transformation. This reaction may make particular use of intermediate heme redox processes at electrochemical interfaces.

Here, we present heme as a bifunctional redox biomolecule with charge transfer and oxygen-shuttling properties that improves the energy efficiency of Li-O_2_ battery systems. We elucidate the mechanism of heme redox during Li-O_2_ reactions by studying electron transfer, oxygen binding and the effect of salt anions on these processes. We find a synergistic effect between the heme molecule and LiClO_4_ that demonstrates the significance of selecting optimal material combinations. As illustrated in Fig. 1b, the electrochemical properties of the heme-assisted Li-O_2_ cell are evaluated to verify the effect on oxygen evolution and cell efficiency. The chemistry and reversibility of discharge products in the cell comprising the heme molecules are investigated by *ex-situ* characterizations. Biomolecules based on similar porphyrin structures can serve as a platform to further improve Li-O_2_ cell efficiency with soluble catalysts. The introduction of eco-friendly biomolecules hints at a simple and sustainable path to develop battery systems that merge biochemistry with advanced energy storage.

## Results

### Heme electrochemistry

**Figure 1:**
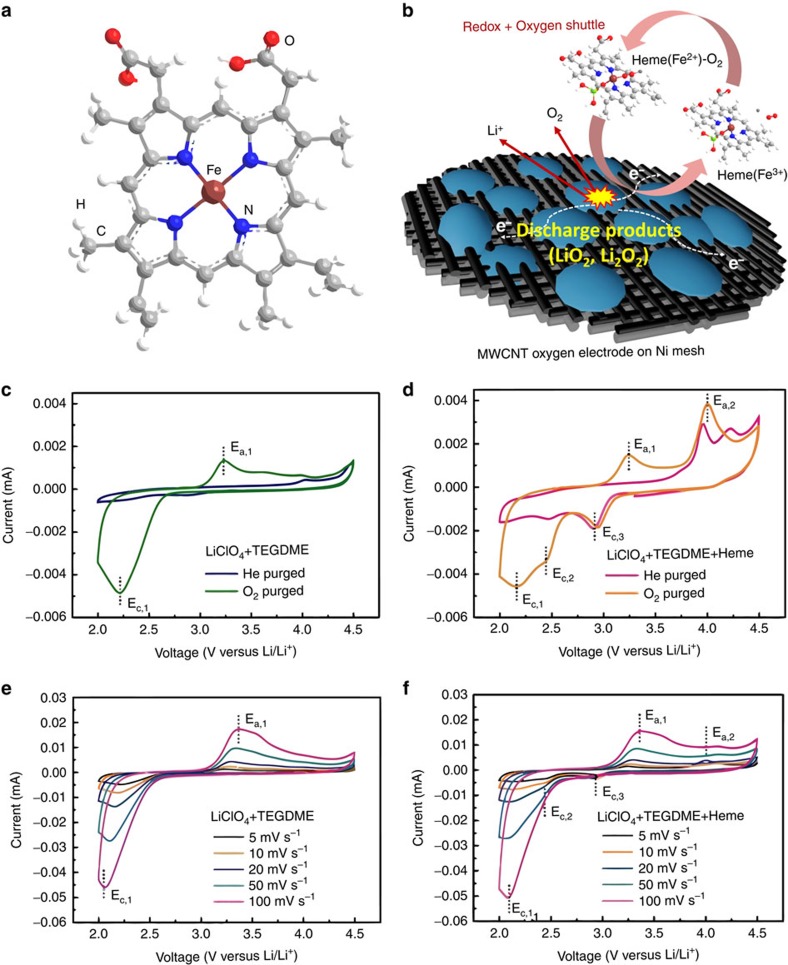
Heme redox properties. (**a**) Structure of heme molecule. (**b**) Schematic illustration of oxygen electrode charged in the heme-containing Li-O_2_ cell; CV curves in various electrolyte media: (**c**) 1 M LiClO_4_+TEGDME and (**d**) 1 M LiClO_4_+TEGDME+Heme after He and O_2_ purging at scanning rate of 5 mV s^−1^. CV curves in the electrolyte of (**e**) 1 M LiClO_4_+TEGDME, (**f**) 1 M LiClO_4_+TEGDME+Heme after O_2_ purging at different scanning rates. All CV curves were collected using glassy carbon electrodes in the voltage window between 2 and 4.5 V. The concentration of heme molecule is 2.3 mM.

To understand the catalytic function of heme for simple electron transfer, oxygen complexation, or a combination thereof, we investigated its electrochemical behaviour in a typical Li-O_2_ cell environment. We show cyclic voltammograms (CVs) to demonstrate the electrochemical properties of hemin (heme with a Cl^−^ anion) dissolved in 1 M LiClO_4_ in tetraethylene glycol dimethyl ether (TEGDME) (Fig. 1c,d). No significant reaction features are seen in the CV curve for LiClO_4_+TEGDME under an inert (He purging) atmosphere (Fig. 1c). However, after O_2_ purging, broad peaks appear in both cathodic and anodic regions near 2.2 V (E_c,1_) and 3.2 V (E_a,1_), respectively (Supplementary Table 1). These peaks correspond to the formation and evolution of Li_2_O_2_ products, respectively^8^,^39^. With the addition of heme, a reduction feature appears even under He purging, indicating that this reaction at 2.94 V (E_c,3_) primarily involves electron transfer (heme(Fe^3+^)+e^−^→heme(Fe^2+^)) when no O_2_ is available (Fig. 1d)^40^. The oxidation feature (E_a,2_) with heme at 3.9 V is likely related to the cathodic reaction (heme(Fe^2+^)→e^−^+heme(Fe^3+^)). We also observe under He purging a peak at 4.2 V, which could relate to the irreversible oxidation of heme(Fe^3+^) to heme(Fe^4+^) in the absence of O_2_ (refs 41, 42). When under an O_2_ atmosphere, additional features are present at 2.5 V (E_c,2_) and 2.2 V (E_c,1_) for the cathodic regime (Fig. 1d). While the 2.2 V (E_c,1_) peak is consistent with O_2_ reduction in the LiClO_4_+TEGDME electrolyte without heme, the feature at 2.5 V (E_c,2_) with heme suggests the formation of an intermediate superoxide: O_2_^−^ or heme(Fe^2+^)–O_2_. We show in the anodic region that features at 3.2 V (E_a,1_) and 4 V (E_a,2_) are present, implying the reverse of the oxygen reduction processes. This result indicates that the heme participates in evolving the superoxide intermediate and facilitating the oxidation of Li_2_O_2_ species.

To individually investigate the heme redox peaks and possible interaction between Li salt and heme molecules, tetraethylammonium perchlorate (TEAClO_4_) was added instead of LiClO_4_ to identify any changes to the electrochemical redox peaks in O_2_ purged solutions (Supplementary Fig. 1). Although the current signal is relatively small due to the low solubility of TEAClO_4_ in TEGDME, we observe no peaks similar to those attributed to the formation and evolution of lithium-oxide products (E_c,1_,E_a,1_) in TEAClO_4_+TEGDME or TEAClO_4_+TEGDME+heme. Nevertheless, redox peaks associated with heme electron transfer (E_c,2_, E_c,3_, E_a,2_) do appear near their expected positions, consistent with CV curves for LiClO_4_+TEGDME+heme. We conclude that Li^+^ cations have no specific interaction with the heme molecule and the heme redox reactions occur independent of Li salt. To disentangle the electrochemical reactions further, we observe the scan rate effect on the electrochemical features (Fig. 1e,f). From the Randles–Sevcik equation, the peak current density is proportional to the square root of the scan rate (*I*_p_=(2.69 × 10^5^)*n*^3/2^ACD^1/2^*v*^1/2^, where *n* is the number of electrons transferred, *A* is the surface area of the electrode, *C* is the concentration of reactant, *D* is the diffusion coefficient of the reactant and *v* is the scan rate)^43^. We find that the peak current of the heme redox at∼3 V (cathodic) and∼4 V (anodic) is less affected by increases in the scan rate than the features related to lithium-oxide product formation and evolution (∼2.3 V (cathodic) and 3.2 V (anodic)). This finding suggests that the O_2_ redox processes are more kinetically limited than those of heme. With a heme concentration of 2.3 mM, significantly less than the concentrations of dissociated LiClO_4_ (1 M) or dissolved O_2_ in TEGDME (4.43 mM)^44^, it is reasonable to attribute the benefit of a small amount of heme to its efficacy as an intermediate in O_2_ redox processes.

### Li-O_2_ battery performance

**Figure 2:**
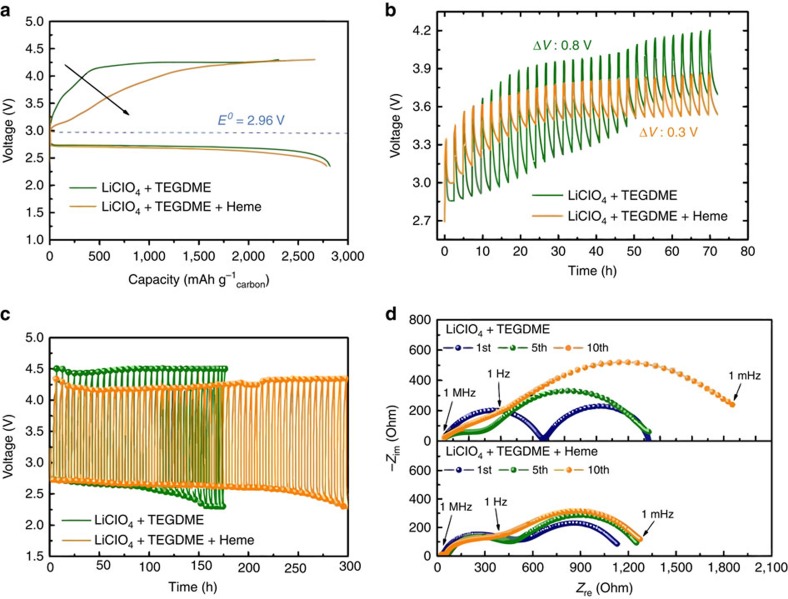
Li-O_2_ cell performance in heme-containing electrolyte. (**a**) Initial charge/discharge curves of the MWCNT electrode in 1 M LiClO_4_+TEGDME and 1 M LiClO_4_+TEGDME+Heme solutions in a voltage window between 4.3 and 2.35 V at a current density of 100 mA g^−1^_carbon_; (**b**) galvanostatic intermittent titration curves of the MWCNT electrode in 1 M LiClO_4_+TEGDME and 1 M LiClO_4_+TEGDME+Heme solutions, which were acquired with a current density of 50 mA g^−1^ for 24 min and a 120 min time interval during the 1st charging; (**c**) Voltage versus time curves of the MWCNT electrodes in 1 M LiClO_4_+TEGDME+Heme solution at various cycles under a specific capacity limit of 600 mAh g^−1^ between 4.5 and 2.3 V at a current density of 200 mA g^−1^_carbon_; (**d**) Electrochemical impedance spectroscopy (EIS) spectra of the MWCNT electrode in 1 M LiClO_4_+TEGDME and 1 M LiClO_4_+TEGDME+Heme solutions after the 1st, 5th, and 10th discharge cycle.

We evaluate the electrochemical functioning of full Li-O_2_ cells with the heme catalyst to assess its practical advantages (Fig. 2). To simplify and focus only on the function of the heme, a multi-walled carbon nanotube (MWCNT) oxygen electrode was used without loading of other catalysts (Supplementary Fig. 2)^14^. We present the first charge/discharge profiles of MWCNT electrodes cycled between 4.35 and 2.35 V at a current density of 100 mA g^−1^_carbon_ in a LiClO_4_+TEGDME electrolyte with and without heme (Fig. 2a). While the cells without and with heme exhibit similar discharge capacities of 2,830 and 2,871 mAh g^−1^_carbon_, the charge capacity of the cell cycled with heme (2,665 mAh g^−1^_carbon_) was higher than that without (2,390 mAh g^−1^_carbon_), corresponding to improved coulombic efficiency (84→93%). In addition, a lower voltage profile was seen in the charging region. To further study the benefit to cell voltage offered by the heme molecule, galvanostatic intermittent titration (GITT) curves during the 1st charge cycle were acquired (Fig. 2b). The difference between constant current voltage (*I*_on_) and open circuit voltage (*I*_off_) in this experiment reflects the overpotential (related to ohmic loss) at each point during charging. Interestingly, the heme-containing electrolyte shows a significantly lower overpotential (∼0.3 V) for charging, compared to the electrolyte without heme (∼0.8 V). While the open circuit voltage in the electrolyte without heme gradually increased, the open circuit voltage in the heme-containing electrolyte quickly reached 3.5 V and was stably maintained for 70 h. The lower charge voltage is related to facile evolution of discharge products promoted by charge transfer between insulating products, heme molecules and the electrode. The charge potential (∼3.5 V) of the Li-O_2_ cell with heme is lower than the CV peak potential (∼4 V) shown in Fig. 1d. The apparent difference of 0.5 V corresponds to an overpotential induced by a high scan rate with CV and by insulating discharge products on the flat glassy carbon disc. A similar voltage difference is evident between the cathodic peak potential in CVs (2.2 V) and the discharge plateau in charge–discharge profiles (2.7 V), which is consistent with the effects of overpotential.

We show the cycle stability of the cells with and without heme between 4.5 and 2.3 V at a current density of 200 mA g^−1^_carbon_ (Fig. 2c). Voltage versus time profiles show the terminal voltages after capacity-limited discharge and charge cycling; a metric related to cell efficiency over multiple cycles. While the terminal charge voltage for a cell without heme quickly reached the top of the voltage window of 4.5 V at the beginning of cycling, a cell with heme exhibited a stable terminal charge voltage of only 4.2 V. For the cell without heme, a voltage drop consistent with cell death was observed after only 150 h. On the other hand, the heme-containing cell maintained its voltage two times longer (∼300 h). These results demonstrate that the heme molecule effectively catalyzes oxygen evolution, improving the efficiency and cycle life of Li-O_2_ batteries.

The electrochemical resistance of a Li-O_2_ cell employing the heme catalyst was evaluated with electrochemical impedance spectroscopy after the 1st, 5th and 10th discharge cycles to discern the cause of decreased overpotentials (Fig. 2d). The 1st semicircle in the Nyquist diagram at high frequencies (ca. 1  MHz through 1 Hz) corresponds to the Li anode/electrolyte interface and the 2nd semicircle at low frequencies (ca. 1 Hz through 1 mHz) is associated with the discharge products and oxygen electrode interface^12^,^45^,^46^,^47^. While the low frequency resistance (1,854 Ω) of the oxygen electrode in the cell without heme increased with increasing cycle number, the oxygen electrode with heme exhibited a lower and relatively stable cell resistance (1,273 Ω) over 10 cycles. The increase of the 2nd semicircle for the cell without heme is likely related to the incomplete evolution and consequent accumulation of discharge products. Continuous and efficient oxygen evolution assisted by heme molecules prevents a substantial increase in cell resistance, consistent with the cycle performance data shown in Fig. 2c.

### Electrochemical product characterization

**Figure 3:**
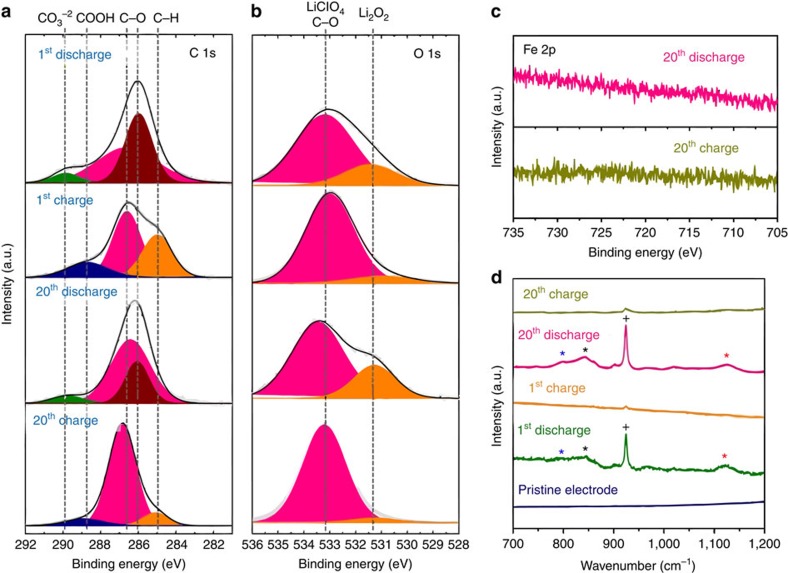
*Ex-situ* measurements on oxygen electrodes. *Ex-situ* X-ray photoelectron spectra obtained from 1 st discharged, 1st charged, 20th discharged, and 20th charged electrodes collected in the (**a**) C 1*s*, (**b**) O 1*s*; (**c**) *Ex-situ* X-ray photoelectron spectra obtained from 20th discharged and 20th charged electrodes collected in the Fe 2*p*; (**d**) E*x-situ* Raman spectra obtained from pristine, 1st discharged, 1st charged, 20th discharged, and 20th charged electrodes. (blue star: Li_2_O_2_, black star: TEGDME, black cross: LiClO_4_, red star: LiO_2_).

*Ex-situ* characterizations were performed to confirm the chemistry of discharge products formed on the electrode surface and verify the reversibility of heme-containing cells. We present *ex-situ* X-ray photoelectron spectra (XPS) of electrode surfaces collected in the C 1*s*, O 1*s*, Fe 2*p* regions (Fig. 3a–c, respectively). C–H and COOH features related to pristine MWCNT and seen in pristine electrodes (Supplementary Fig. 3), were diminished after discharge and retrieved after charge, consistent with the formation and subsequent evolution of a layer of discharge products (Fig. 3a). Li_2_O_2_ products were detected on discharged electrodes and the related feature (531.4 eV) was indeed diminished after charging (Fig. 3b). Product chemistry was similarly examined by the *ex-situ* Raman spectroscopy of these electrodes (Fig. 3d). After discharge, both LiO_2_ and Li_2_O_2_ species were detected at 1,121 and 791 cm^−1^, respectively^14^,^48^,^49^,^50^,^51^, and these product species appear to be fully reversible. Features related to CO_3_^2−^ and C–O are apparent on several electrodes, but these likely result from carbon electrode degradation^52^, electrolyte decomposition^53^ or minor surface contamination by limited exposure to ambient air. In the O 1*s* XPS spectrum, strong peaks related to LiClO_4_ salt were found for all samples, suggesting that the electrolyte is integrated into the electrochemical products. The products on discharged electrodes show no XRD peaks verifying that the lithium-oxide species are not well crystallized (Supplementary Fig. 4). To investigate the possibility that the heme molecule could be incorporated into the discharge products, we show the Fe 2*p* XPS region of a discharged and charged electrode after 20 cycles (Fig. 3c). We observe no Fe peaks from either electrode, demonstrating that the heme molecule only facilitates the Li-O_2_ cell reaction as a soluble catalyst and does not form a component of the product as a result of heme decomposition and side reactions. Heme components were also not observed on discharged or charged electrodes using *ex-situ* Raman spectroscopy (Supplementary Fig. 5).

We show the surface morphologies of the MWCNT electrodes loaded on Ni-mesh at various charge–discharge states (1st and 20th cycles). (Supplementary Fig. 6) Cell tests were performed in 1 M LiClO_4_+TEGDME+Heme solutions to collect electrodes at different electrochemical states for *ex-situ* SEM analysis. When the cells were first discharged, discharge products were precipitated and covered the electrode (Supplementary Fig. 6a,b). When cells were subsequently charged, the products were decomposed and disappeared from the electrode (Supplementary Fig. 6c). After the 20th cycle, results similar to those of the 1st cycle were observed (Supplementary Fig. 6d,e). These results verify the excellent reversibility of the Li-O_2_ cell employing a heme biomolecular catalyst. From the magnified SEM images (Supplementary Fig. 6f), it can be seen that the products were fully covered on the electrode and the shape looks like a mixture of particulate and amorphous products.

### Heme–oxygen interaction

**Figure 4:**
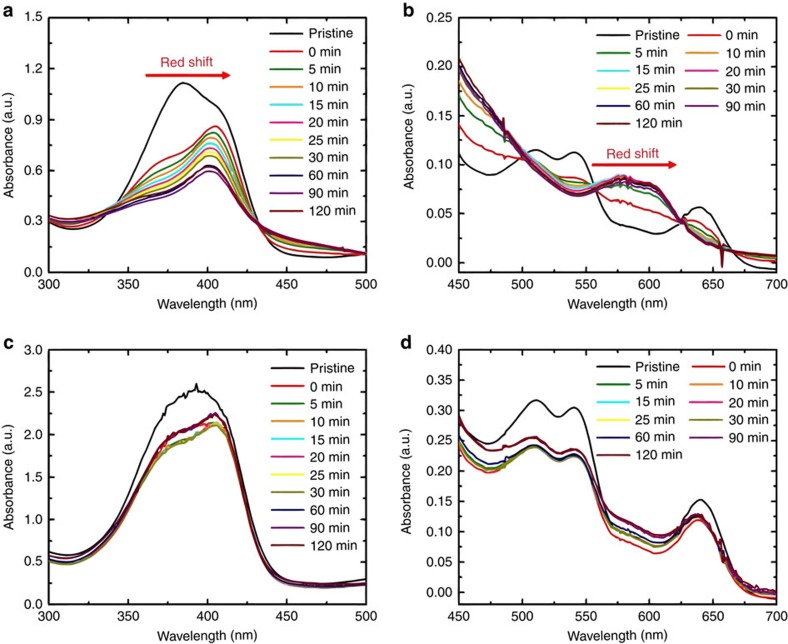
Chemical states of heme molecule by binding with oxygen species. Ultraviolet–vis spectra of heme-containing electrolytes with increasing time after the injection of KO_2_ solution; (**a**) TEGDME+Heme (Soret band region), (**b**) TEGDME+Heme (Q-band region), (**c**) 1 M LiClO_4_+TEGDME+Heme (Soret band region), (**d**) 1 M LiClO_4_+TEGDME+Heme (Q-band region).

We utilized ultraviolet–vis absorption to explore how the chemical states of the heme molecule change in the presence of oxygen (Fig. 4). Potassium oxide (KO_2_), which generates the superoxide ion (O_2_^−^) in solution, was introduced into various electrolyte media and the absorbance of the heme was monitored over time. No particular absorbance feature was observed for TEGDME and LiClO_4_+TEGDME solutions in the visible range, but a feature <300 nm is indicative of TEGDME (Supplementary Fig. 7). Superoxide is known to absorb at ∼253 nm and a modest increase in the absorbance at <300 nm after injection of KO_2_ indicates its presence^54^,^55^. When the heme molecule is introduced into the solvent, a strong peak near 400 nm (Soret band) and sub-peaks between 450 and 700 nm (Q-bands) were identified, corresponding to typical heme absorbance (Fig. 4; Supplementary Table 2)^56^. The Soret band indicates a strong electronic transition of the metalloporphyrin and the Q-band is associated with a weak transition in the porphyrin ring. For a heme-containing solution without LiClO_4_, a red shift of the Soret and Q-bands were observed after KO_2_ injection (Fig. 4a,b). A red shift of the Soret band of heme molecules has been documented previously as relating to the transition of the Fe ions from high- to a low-spin state due to coordination of species with the Fe *d* orbital (*e*_g_ orbital)^56^,^57^. In the case of the red shift caused by adding KO_2_, this suggests a change in state from heme(Fe^3+^) to heme(Fe^2+^)–O_2_ by coordination with a soluble superoxide^58^. When LiClO_4_ is included in the solution with heme, the addition of KO_2_ to the solution demonstrates a similar red shift in the Soret band, but is stabilized quickly (Fig. 4c,d). In this case, we accept that two phenomena may occur, which prevents further coordination between the heme and the superoxide. First, the presence of Li^+^ may initiate precipitation of LiO_2_ or disproportionation to form Li_2_O_2_ so that superoxide is quickly removed from solution. Second, the ClO_4_^−^ anion may competitively bind to the heme(Fe^3+^) site due to its negative charge and delocalized electron. There is evidence for anion coordination from the slight red shift (384→394 nm) in the Soret band with LiClO_4_ even before KO_2_ injection. To investigate the Li salt anion effect on coordination chemistry, experiments were repeated with LiPF_6_ salt (Supplementary Fig. 8). We find that the PF_6_^−^ anion does not influence the Soret band and Q-band of heme when KO_2_ is added. Thus, we suggest that the superoxide cannot bind with heme in this electrolyte environment. There is some evidence to support the decomposition of PF_6_^−^ by the superoxide^59^, which could prevent heme-superoxide coordination. In addition, we note the potential effect of a higher donor number for the ClO_4_^−^ anion (8.4) compared with PF_6_^−^ (2.5), which may help to stabilize superoxide species and form a superoxide complex with heme molecules^60^.

### Heme structural change during reaction

**Figure 5:**
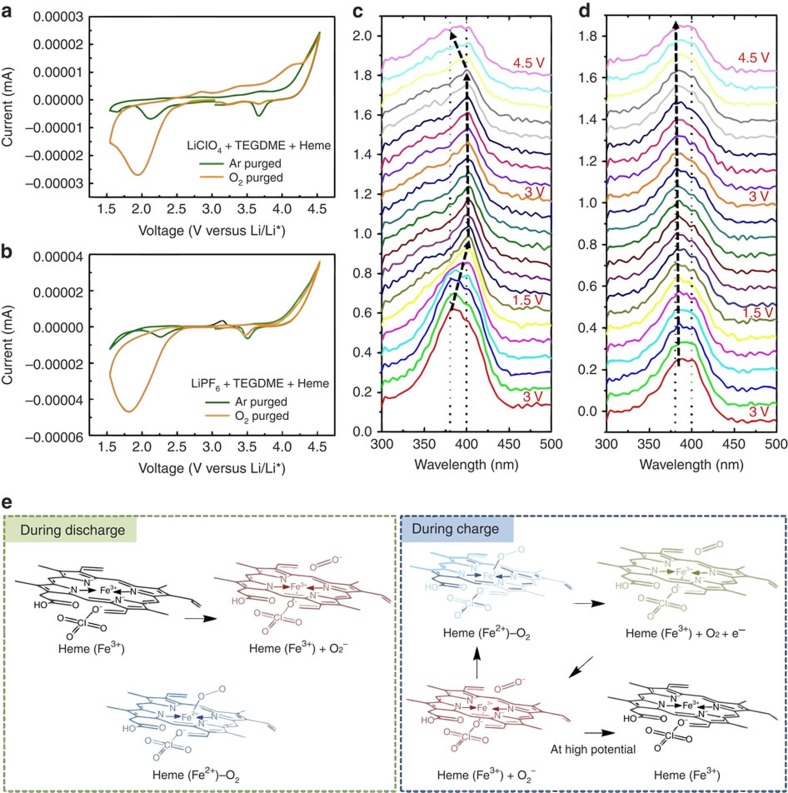
*In situ* observation of heme molecule at different electrochemical states. Spectroelectrochemical data of the different electrolytes (1 M LiClO_4_+TEGDME+Heme and 1 M LiPF_6_+TEGDME+Heme); CV curves of (**a**) 1 M LiClO_4_+TEGDME+Heme and (**b**) 1 M LiPF_6_+TEGDME+Heme and corresponding ultraviolet–vis spectrum of (**c**) 1 M LiClO_4_+TEGDME+Heme and (**d**) 1 M LiPF_6_+TEGDME+Heme. The spectroelectrochemical tests were performed with a commercial Au honeycomb working electrode and Au counter electrode in the voltage range of −1.5 to 1.5 V versus Pt pseudo-reference in an O_2_ atmosphere. Voltage values were converted to reflect a Li/Li^+^ reference; (**e**) Schematic illustration of chemical states of the heme molecule in Li-O_2_ cell.

To better elucidate chemical changes in the heme molecule at different electrochemical states during electroreduction and oxidation, we conducted *in situ* spectroelectrochemical measurements on a heme-containing electrolyte. We present CV curves (Fig. 5a,b) and the corresponding ultraviolet–vis spectra collected at different voltages (Fig. 5c,d). The spectroelectrochemical cells were designed with symmetric Au working and counter electrodes. While additional redox peaks related to Au oxidation/reduction near 3.67 V were found, the CV features (Fig. 5a) are similar to those in Fig. 1d, which was obtained with a glassy carbon working electrode, Pt counter electrode and Li/Li^+^ reference electrode. For an Ar purged cell, the heme Soret band exhibits a red shift during the cathodic scan and its position is maintained for the reverse scan up to 4.5 V versus Li/Li^+^ (Supplementary Fig. 9). As we previously observed in CV results, this shift corresponds to direct electron transfer to heme(Fe^3+^) without oxygen (heme(Fe^3+^)+e^−^→ heme(Fe^2+^)). In an O_2_ atmosphere, a red shift is similarly evident following reduction, indicating the formation of either heme(Fe^2+^) or heme(Fe^2+^)–O_2_^61^. A blue shift at 4.2 V occurs during oxidation, highlighting the reversibility of the reduction process in the presence of O_2_ (Fig. 5c; Supplementary Fig. 10). The heme is therefore thought to bind O_2_^−^ ions during the discharge reaction and release O_2_ and e^−^ (to the electrode) during the charge reaction (Fig. 5e). On charging near 3.9 V, any existing O_2_^−^ ions, potentially generated from oxide products (LiO_2_ or Li_2_O_2_) are bound with available heme(Fe^3+^), itself regenerated by heme(Fe^2+^)–O_2_ oxidation (Fig. 5e). The heme(Fe^2+^)–O_2_ complex is mobile in the electrolyte, unlike Li-O_2_ solid products, and thus may travel to and from the electrode surface. When most oxide products are decomposed, the heme(Fe^2+^) molecules are oxidized to heme(Fe^3+^) themselves at high anodic potential. Redox mediation by heme therefore involves a complexation mechanism, unlike conventional mediators, which only function by electron transfer.

## Discussion

Li-O_2_ cell function with heme can be summarized by some representative steps for discharge and charge (Fig. 5e). Our findings suggest a reaction mechanism involving the coordination of heme molecules during cell operation:

### Oxygen reduction reactions (discharge)


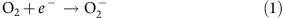



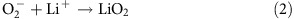



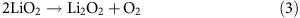



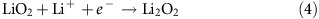



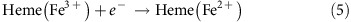



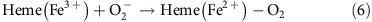


### Oxygen evolution reactions (charge)


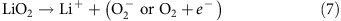



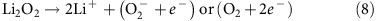



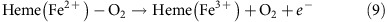



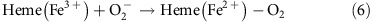



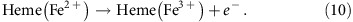


During the discharge reaction, superoxides (O_2_^−^) are generated by oxygen reduction (equation (1)) and they form lithium oxides such as LiO_2_ and Li_2_O_2_ (equations (2, 3 and 4)). Heme (Fe^3+^) may accept an electron from the electrode or bind with available O_2_^−^, forming heme(Fe^2+^) or the heme(Fe^2+^)–O_2_ complex (equations (5 and 6)). On charging, the heme(Fe^2+^)–O_2_ complex evolves oxygen and transfers an electron directly to the electrode (redox mediator), thereby reverting back to the heme(Fe^3+^) state (equation (9)). Heme(Fe^3+^) may accept any available superoxides generated from the oxidation of LiO_2_ or Li_2_O_2_ (equations (7 and 8)) and form the heme(Fe^2+^)–O_2_ complex once again (equation (6)). These charge processes may circulate until most of the available oxide species has been evolved. At high potential where Li-O_2_ products are fully evolved, the heme(Fe^3+^) remains in its original state (equation 10). In this scheme, we may consider the heme molecule both a ‘sink' and ‘transporter' for superoxide and electrons during charging. Its influence on the kinetics of LiO_2_ or Li_2_O_2_ evolution makes the designation of soluble catalyst appropriate.

Interestingly, the heme molecule exhibits this redox function in a LiClO_4_ containing electrolyte but not one with LiPF_6_. We carried out spectroelectrochemical measurements in a LiPF_6_ containing electrolyte with the same concentration of heme to verify the Li salt effects (Fig. 5b,d). Although a large reduction peak in the CV near 1.8 V corresponds to discharge product formation there are no oxidation peaks in the charge region. The ultraviolet–vis spectra indicate no significant shifts in the Soret band during reduction or oxidation with a LiPF_6_ electrolyte, demonstrating that the heme chemical structure is not changed even in an O_2_-containing environment (Fig. 5d). This result is consistent with the time-course ultraviolet–vis experiment as shown in Supplementary Fig. 8 and provides additional evidence that PF_6_^−^ anions not only inhibit oxygen coordination but also prevent electron transfer with heme. Together, these results indicate that the heme undergoes several charge transfer processes including electron transfer, O_2_ coordination and anion inhibition. We highlight the need to select appropriate combinations of electrolyte and redox biomolecule to achieve electrochemical synergies.

In summary, we report the use of heme as an abundant and eco-friendly biomolecular catalyst to facilitate Li-O_2_ oxidation and improve battery function. The soluble heme molecule enables charge transfer between insulating Li-O_2_ discharge products and the electrode by engaging in electron transfer and coordinating with superoxide intermediates. The oxygen species bind with the Fe^3+^ heme centre and release oxygen while transferring charges to the electrode. The reversible chemical transitions of the heme under various electrochemical conditions were investigated by *in situ* spectroelectrochemical measurements. Interestingly, the heme molecules exhibit their function synergistically with ClO_4_^−^ anions in the electrolyte and not with PF_6_^−^, indicating that pairing the Li salt and redox molecules is necessary to promote effective catalytic function. The Li-O_2_ cell with heme catalysis achieves a lower polarization and longer cycle life, compared with the control. We also verify the reversible formation and decomposition of LiO_2_ and Li_2_O_2_ on the oxygen electrode by *ex-situ* characterization and show that heme is not incorporated into solid products, but remains a mobile electrolyte species. Indeed, redox biomolecules with complexing catalytic functions present a new path to improve electrochemical storage efficiency using sustainable materials.

## Methods

### Materials and chemicals

High purity multi-walled carbon nanotubes (MWCNT, SWeNT SMW100) were donated by SouthWest Nanotechnologies. Hemin (from bovine, ≥90%), lithium perchlorate (LiClO_4_, battery grade, 99.99%), tetraethylene glycol dimethyl ether (TEGDME, anhydrous, ≤80 p.p.m. H_2_O), tetraethylammonium perchlorate (TEAClO_4_, for electrochemical analysis, ≥99%), poly(vinylidene fluoride) (PVDF, Mw ∼180,000) and 1-methyl-2-pyrrolidinone (NMP, anhydrous, 99.5%) were purchased from Sigma-Aldrich (St Louis, USA). Potassium superoxide (KO_2_, 96.5%) was purchased from Alfa Aesar (Ward Hill, USA). All chemicals were used without further purification.

### Electrochemical characterization

Electrochemical characterization was performed in sealed purged Swagelok cells with gas inlet and outlet valves on the oxygen electrode side. The oxygen electrode was fabricated by casting a slurry, consisting of 90 wt% of oxygen electrode materials (MWCNT) and 10 wt% PVDF binder in NMP solvent, on a flattened Ni-mesh (diameter: 12.7 mm). The electrode was dried under vacuum at 75 °C before cell assembly in an argon-filled glove box. For electrolyte preparation, LiClO_4_ was dried for 24 h in a vacuum oven and then stored in the glove box for use. The electrolytes were prepared by mixing the LiClO_4_ (1 M), TEGDME and hemin (2.3 mM) in the Ar glove box and stirring overnight. Li-metal foil (11.1 mm diameter) was used as the counter electrode and the separator was a Whatman GF/A glass fibre (13 mm diameter). To eliminate the concentration effect of heme in electrolyte, the 2.3 mM concentration of heme we have used in this study is close to the solubility limit in TEGDME. A Bio-Logic VSP potentiostat with impedance function was used for galvanostatic cycling, galvanostatic intermittent titration and electrochemical impedance spectroscopy measurements of cells. All electrochemical experiments were performed at room temperature.

### Cyclic voltammetry tests

For CV experiments, a three-electrode cell was used with 50 ml of electrolytes and a sealed Li/Li^+^ reference electrode. All electrolytes and fresh reference electrodes were prepared inside an isolated glove box. Due to low solubility of the TEAClO_4_ salt in TEGDME, the solution was centrifuged after stirring to remove residual undissolved salts. TEAClO_4_ containing electrolytes are considered to be saturated solutions. A Pt mesh and a 5 mm diameter glassy carbon electrode were used as the counter electrode and the working electrode, respectively. On removal from the glove box, solutions were immediately purged with dry He gas for 30 min to ensure an inert atmosphere. CV experiments were carried out under an He blanket in a voltage window between 2 and 4.5 V at different scan rates. Solutions were then purged with dry O_2_ for 30 min and CV experiments were repeated. The glassy carbon electrode was cleaned with ethanol and acetone after each CV test and dried completely.

### Time-course ultraviolet–vis spectroscopy tests

The concentration of hemin molecules was diluted to 0.38 × 10^−4^ M in TEGDME and 1 M LiClO_4_/TEGDME for time-course ultraviolet–vis spectra. Overall, 5 mM KO_2_ was mixed with TEGDME for 24 h in an Ar-filled glove box to saturate the precursor solution. The actual concentration of KO_2_ in TEGDME is <5 mM due to reaction with residual water and incomplete dissolution. Electrolyte solutions (2.5 ml) were added into a quartz cuvette and the cuvettes were tightly sealed with a septum cap. A syringe injected 0.5 ml of the KO_2_ supernatant into the cuvettes and ultraviolet–vis spectra were measured for 2 h (Varian Cary 3E, Agilent Technologies, USA). The nominal ratio of KO_2_ to hemin in the mixture is ∼27:1, but due to the poor solubility of KO_2_ in TEGDME, the true ratio is significantly less.

### Spectroelectrochemical measurement

The spectroelectrochemical measurement was carried out to monitor the chemical change of the heme molecule during discharge and charge. The electrolytes were transferred into a quartz cuvette with a commercial Au honeycomb electrode (Pine Research Instrumentation, NC, USA). The working electrode is perforated with a honeycomb pattern of holes that allow light to pass through the electrode. The active surface of the working electrode includes Au coating along the inner walls of the holes. As the light beam from the spectrometer passes through the holes, the beam grazes the walls of each hole. An Au counter electrode and Pt pseudo-reference electrode were used to construct the 3-electrode cell. The electrode potential was controlled with a potentiostat (Princeton Applied Research, VersaSTAT 4) and ultraviolet–vis spectra were collected at varying potentials on a Varian Cary 50. The electrolytes were purged with dry Ar or dry O_2_ for at least 20 minutes before each experiment. All potential values for CVs were calibrated by voltage versus Li/Li^+^ (*E*°=−3.04 V).

### *Ex-situ* characterization

The crystal structures and bonding within the electrode samples after cycling were analyzed by X-ray photoelectron spectroscopy (XPS), Raman spectroscopy (T64000, Horiba Jobin-Yvon, excitation at 633 nm) and X-ray diffraction (XRD, RIGAKU, D/MAX-RC). Raman spectra were recorded with a CCD detector that was cooled to −70 °C and a silicon wafer was used to calibrate the Raman shift. XPS measurements were performed at Brookhaven National Laboratory on a SPECS GmbH instrument under ultrahigh vacuum (UHV) conditions. The X-ray source was Al Kα at a power of 300 W.

### Data availability

The data that support the findings of this study are available from the corresponding author upon request.

## Additional information

**How to cite this article:** Ryu, W.-H. *et al*. Heme biomolecule as redox mediator and oxygen shuttle for efficient charging of lithium-oxygen batteries. *Nat. Commun.* **7,** 12925 doi: 10.1038/ncomms12925 (2016).

## Supplementary Material

Supplementary Information Supplementary Figures 1-10 and Supplementary Tables 1-2
