# Supplementary material for: Heme biomolecule as redox mediator and oxygen shuttle for efficient charging of lithium-oxygen batteries
Source: Nat Commun. 2016 Oct 19;7:12925. doi: 10.1038/ncomms12925 (PMC5075788; doi:10.1038/ncomms12925)
Supplement: Supplementary Information — Supplementary Figures 1-10 and Supplementary Tables 1-2 [file ncomms12925-s1.pdf]

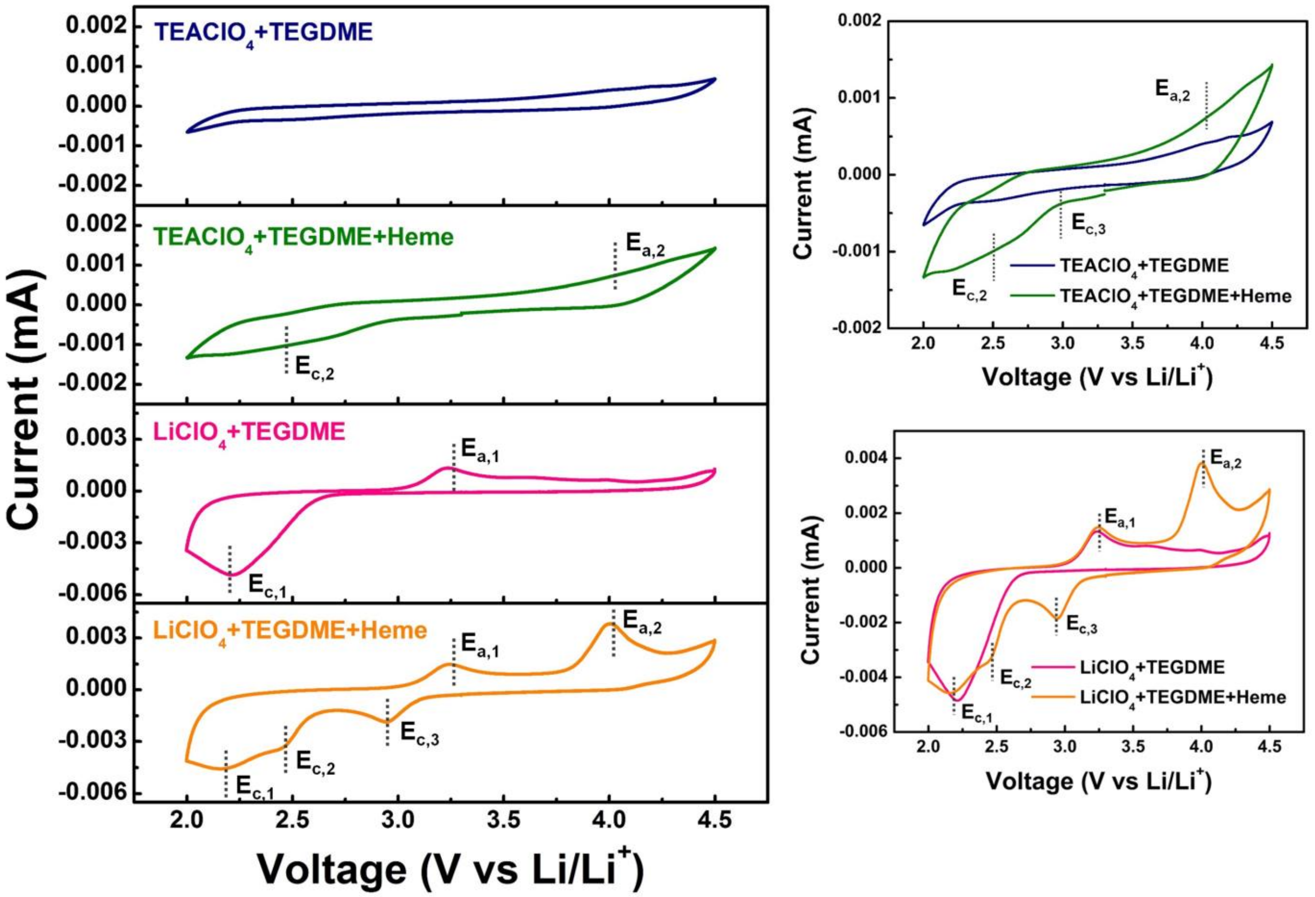

Supplementary Figure 1. Cyclic voltammetry (CV) curves in various media; $TEAClO_4$+TEGDME, $TEAClO_4$+TEGDME+Heme, $LiClO_4$+TEGDME, $LiClO_4$+TEGDME+Heme after O_2 purging at scan rate of 5 mV s^{-1}. All CV curves were collected using a glassy carbon electrode in the voltage window between 2.0 and 4.5 V. The concentration of hemin molecule is 2.3 mM.

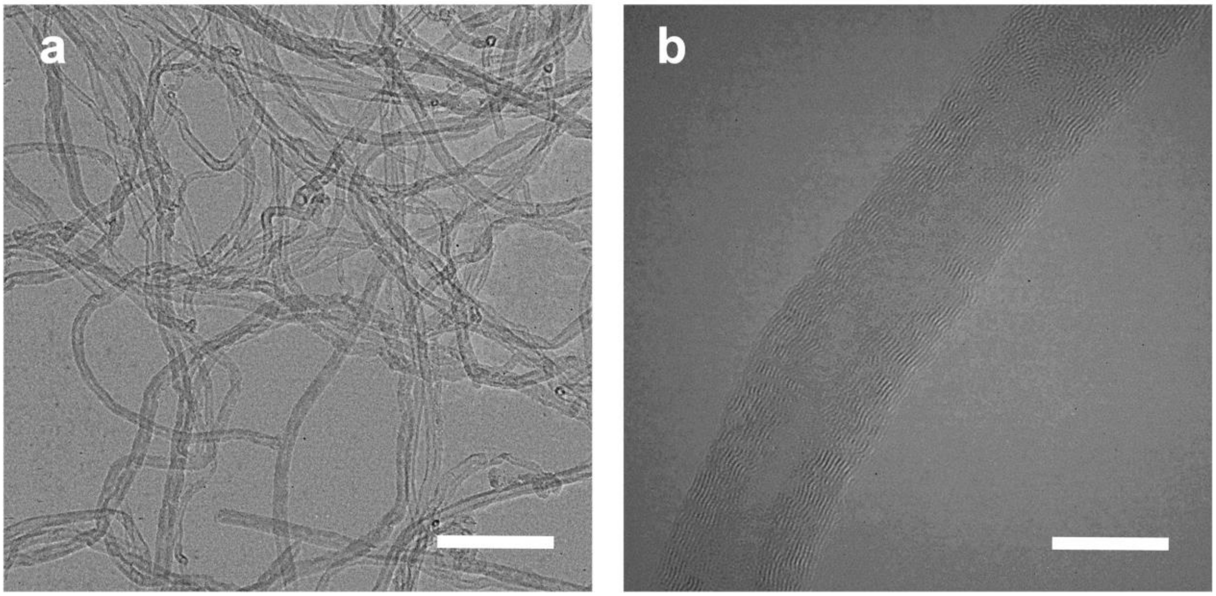

Supplementary Figure 2. TEM images of the 1-D MWCNT with (a) low and (b) high magnifications. The average diameter of MWCNTs is about 15 nm. Scale bars represent in (a) 100 nm and (b) 10 nm.

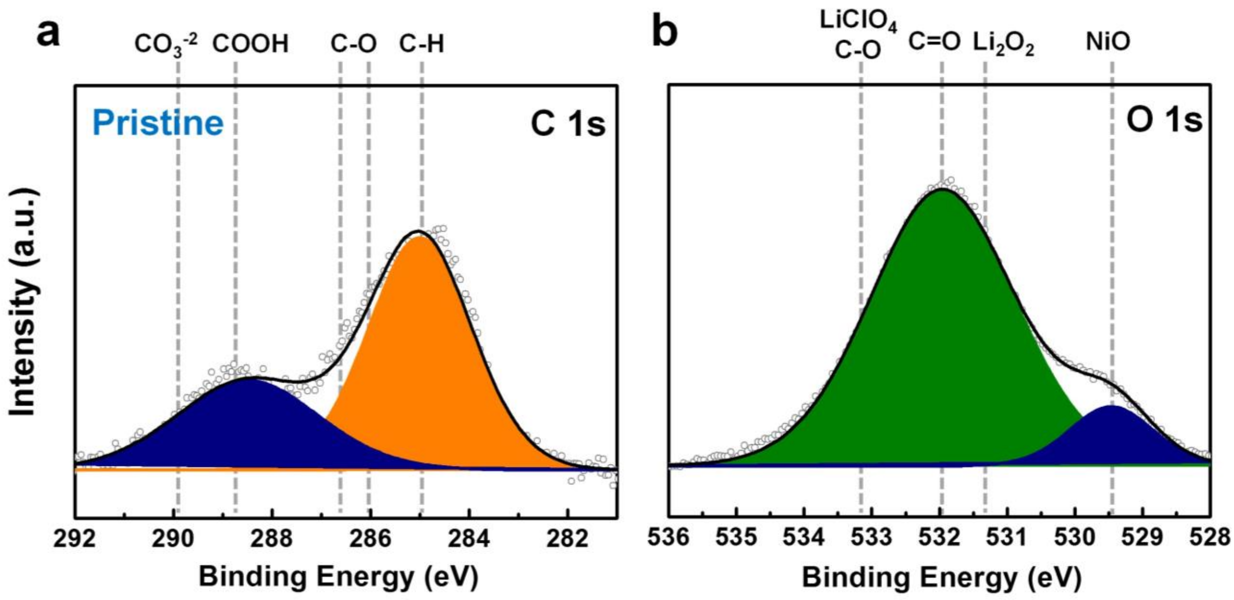

Supplementary Figure 3. X-ray photoelectron spectra obtained from pristine MWCNT electrodes collected in the (a) C 1s and (b) O 1s.

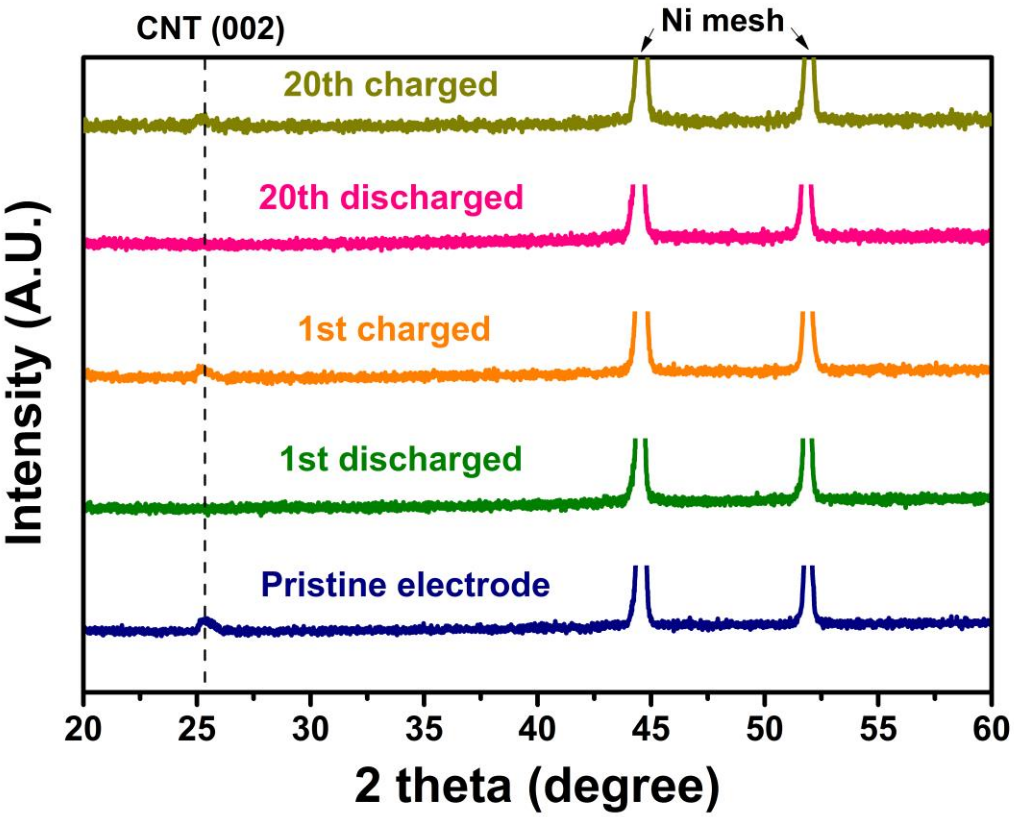

Supplementary Figure 4. *Ex situ* XRD data of pristine, 1st discharged, 1st charged, 20th discharged, and 20th charged electrodes. There is no product peak such as Li_2O_2 near 33° and 35°. The (002) peak of CNT disappeared after discharge due to covering of products on the CNT surface, and the peak was reversibly retrieved after each charge states.

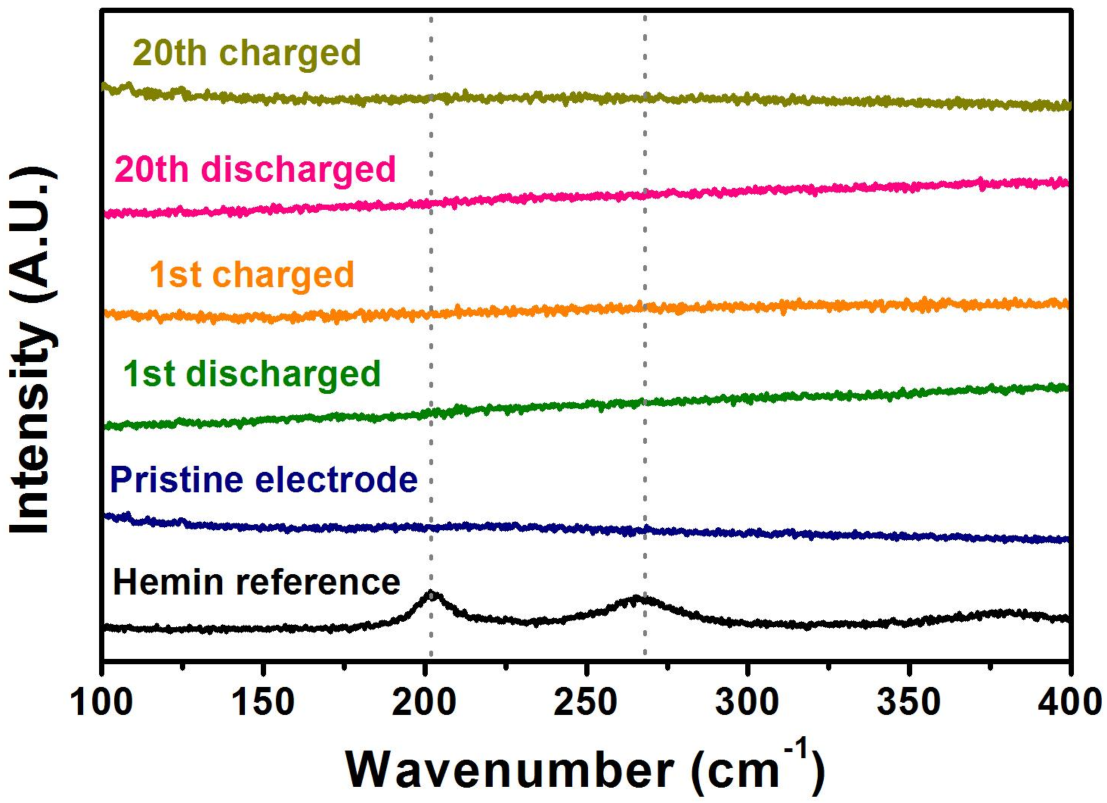

Supplementary Figure 5. *Ex situ* Raman spectra obtained from a hemin reference powder, pristine electrode, 1st discharged, 1st charged, 20th discharged, and 20th charged electrodes. No peak related to the heme molecule is found in the electrode spectra, indicating that heme is not incorporated into the discharge products.

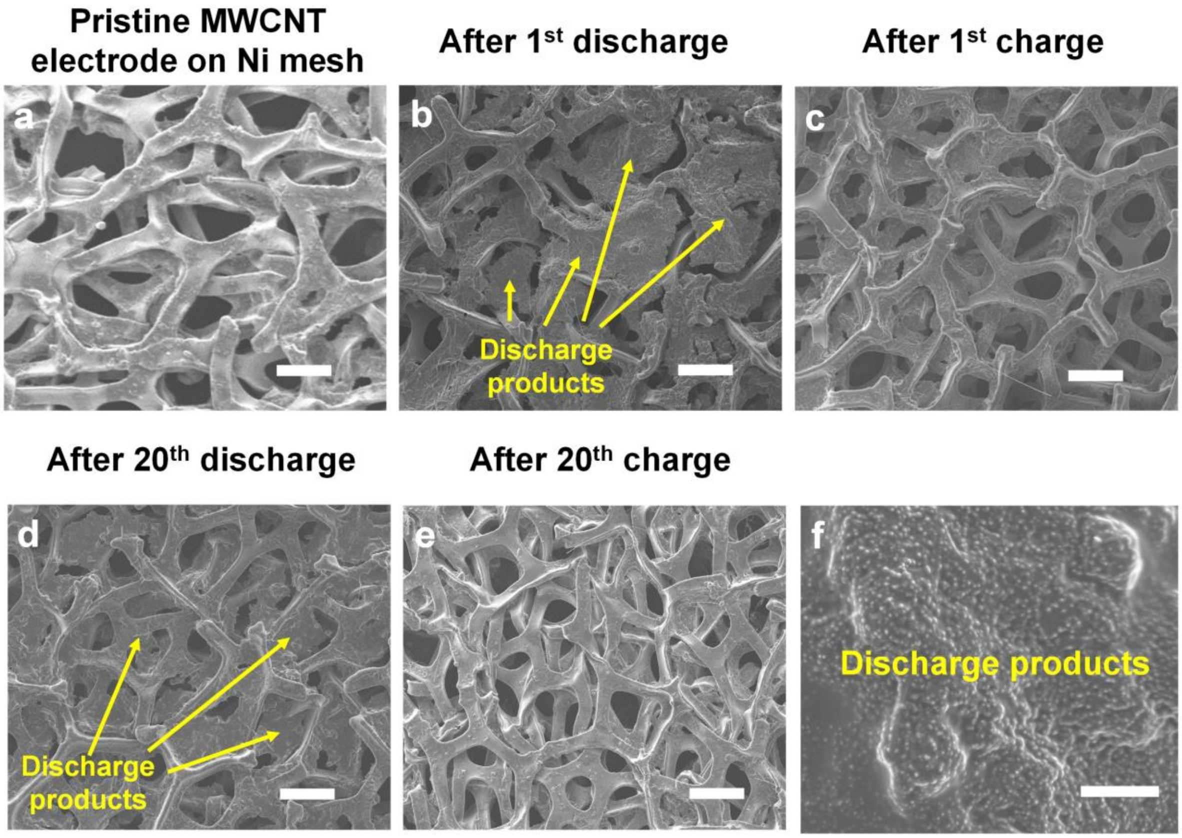

Supplementary Figure 6. Surface morphologies of the (a) pristine MWCNT electrode on Ni mesh, (b) MWCNT electrode after 1st discharge, (c) MWCNT electrode after 1st charge, (d) MWCNT electrode after 20th discharge, and (e) MWCNT electrode after 20th charge. Cell tests were performed in 1 M $LiClO_4$+TEGDME+Heme solutions to collect electrodes at different electrochemical states for ex-situ SEM analysis.; (f) Magnified SEM image of the discharge products collected from the MWCNT electrode after 1st discharge. (Specific capacity limit: 600 mAh g^{-1}) Scale bars represent in (a-e) 200 μm and (f) 10 μm.

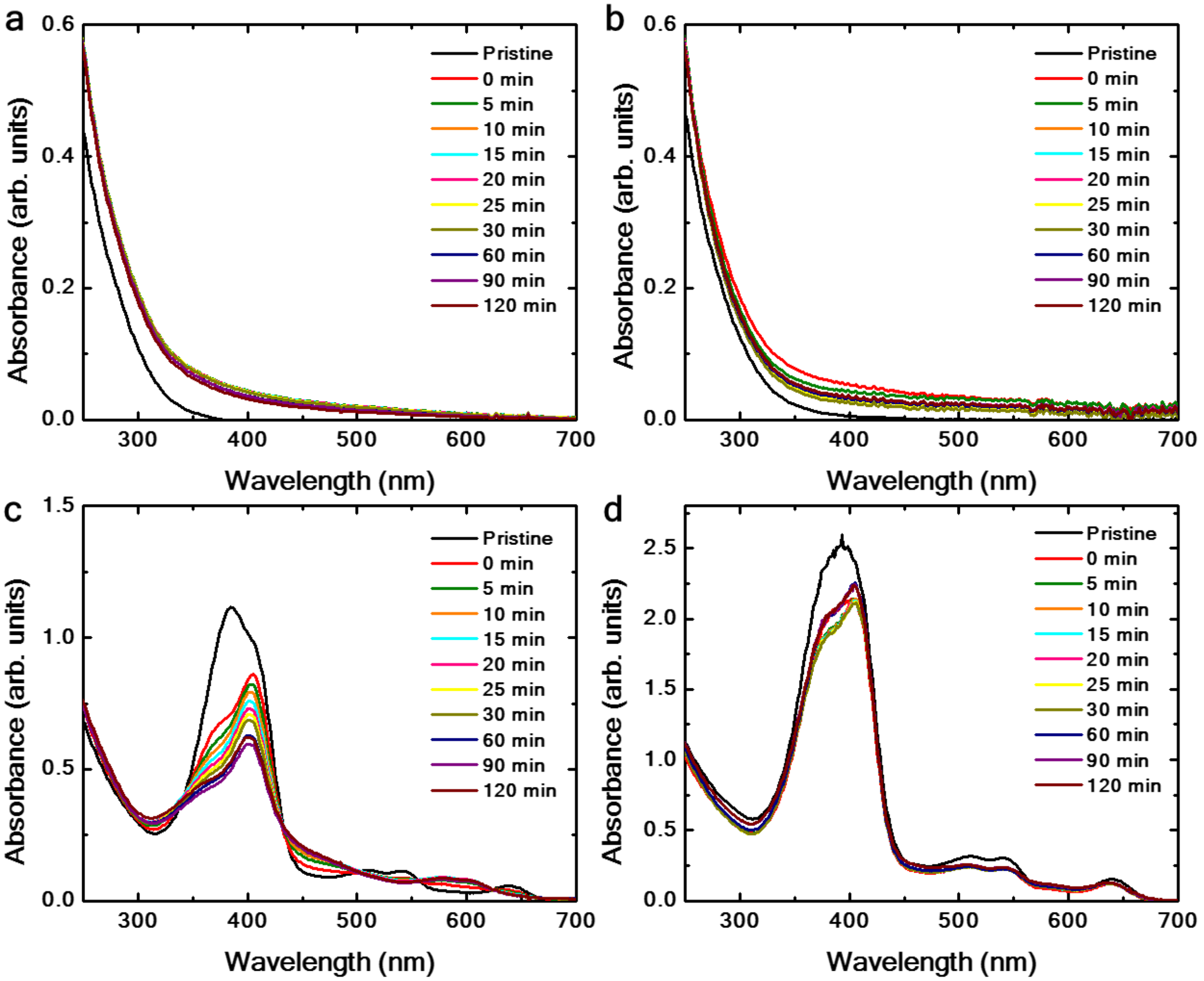

Supplementary Figure 7. UV-vis spectra of heme-containing electrolytes with increasing time after the injection of KO_2 solution; (a) TEGDME, (b) 1 M $LiClO_4$+TEGDME, (c) TEGDME+Heme, (d) 1 M $LiClO_4$+TEGDME+Heme.

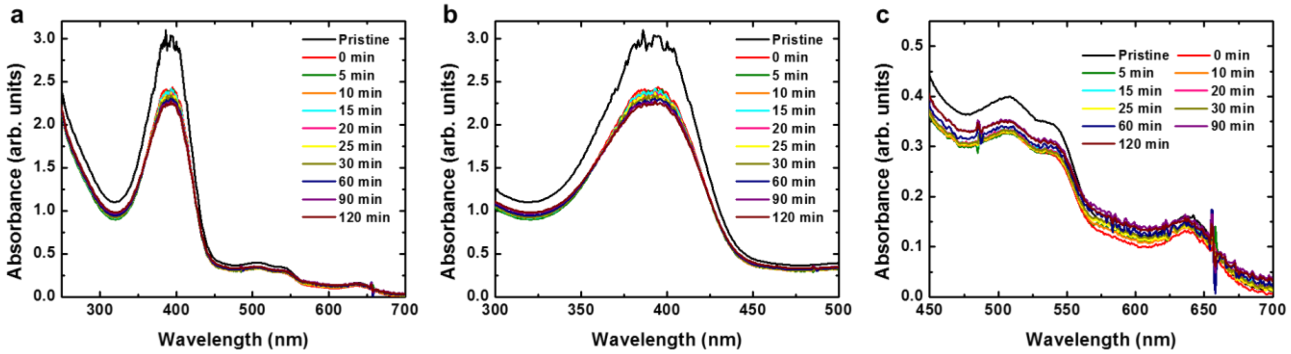

Supplementary Figure 8. UV-vis spectra of the $LiPF_6$+TEGDME+Heme electrolytes with increasing time after the injection of KO_2 solution; (a) full spectra, (b) Soret band region, and (c) Q-band region.

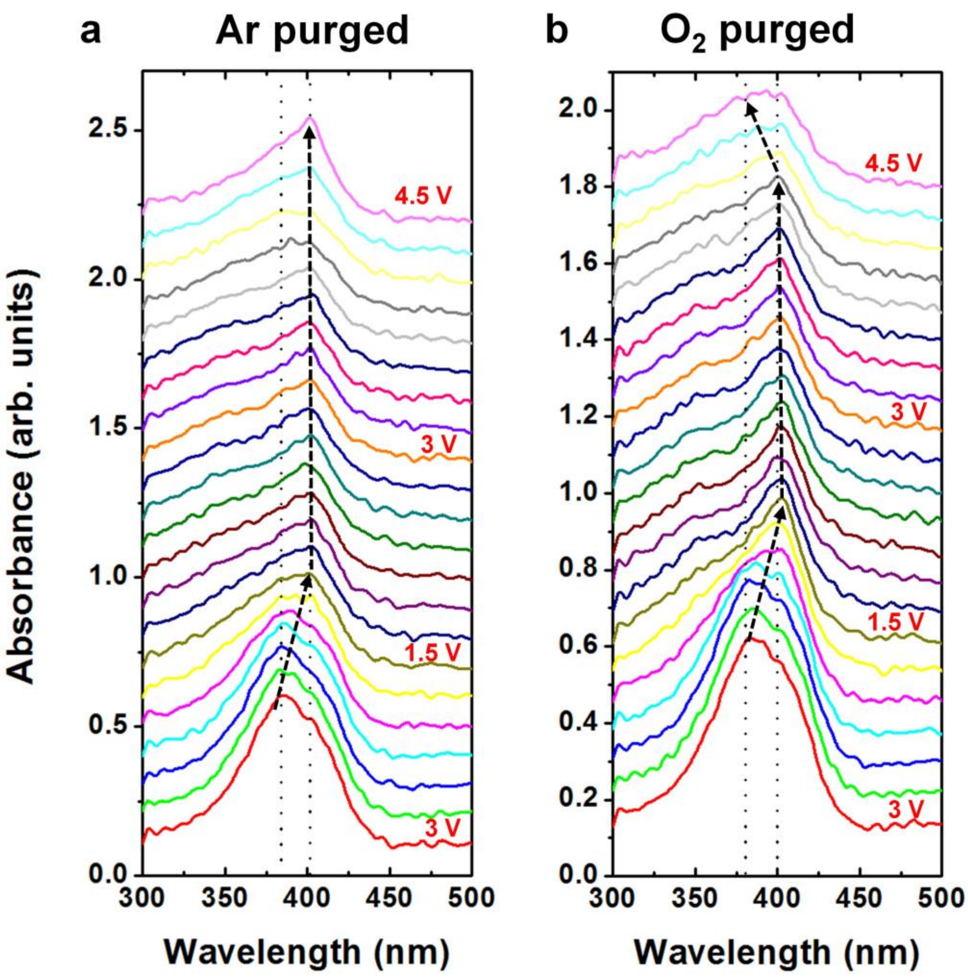

Supplementary Figure 9. Spectroelectrochemical data of the 1 M $LiClO_4$+TEGDME+Heme corresponding to CV profiles in Figure 5a collected after (a) Ar and (b) O_2 purging. The spectroelectrochemical tests were performed in a voltage range of -1.5 to 1.5 V versus a Pt pseudo-reference. Voltage values were converted to reflect a Li/Li^+ reference. A commercial Au honeycomb electrode which allow light to pass through the electrode consists of working and counter electrodes.

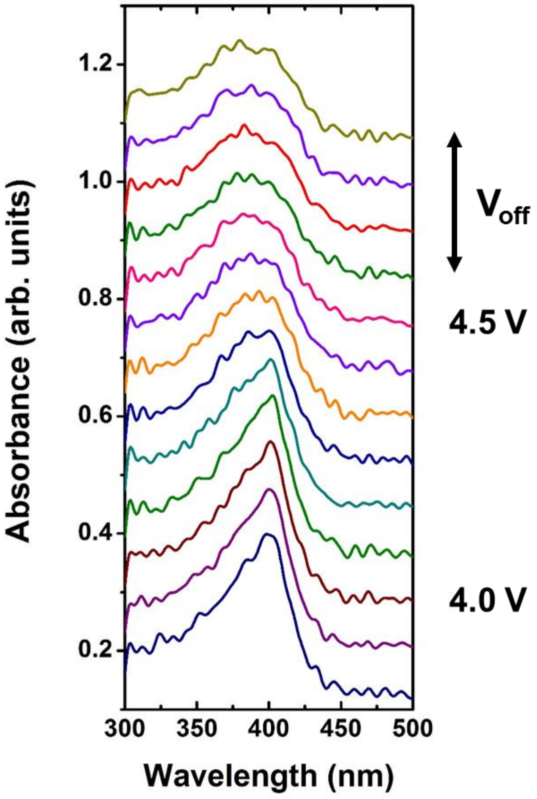

Supplementary Figure 10. Spectroelectrochemical data of 1 M $LiClO_4$+TEGDME+Heme for charge region. The additional UV-vis spectrums were collected after charge to confirm the heme chemical states.

Electrolyte	$E_{c,1}$ (V)	$E_{c,2}$ (V)	$E_{c,3}$ (V)	$E_{a,1}$ (V)	$E_{a,2}$ (V)
**$LiClO_4$**	2.2			3.2	
**$LiClO_4$ + Heme**	2.2	2.5	2.94	3.2	4.0

Supplementary Table 1. Voltammetric properties of oxygen saturated $LiClO_4$ + TEGDME electrolytes without and with heme molecule (Scan Rate: 5 mV s^{-1})

Electrolyte	Soret band (nm)	Q band (nm)	
**Heme (pristine)**	384	510	541
**Heme (120 min.)**	401	580	605
**$LiClO_4$ + Heme (pristine)**	394	510	541
**$LiClO_4$ + Heme (120 min.)**	404	510	541

Supplementary Table 2. Peak wavelength values of TEGDME+Heme and $LiClO_4$+TEGDME+Heme solutions at Soret band and Q-band. The values are obtained from UV-vis spectra of Figure 4.
